# Correlation of N-Acetyltransferase 2 Genotype with Isoniazid Acetylation in Polish Tuberculosis Patients

**DOI:** 10.1155/2013/853602

**Published:** 2013-12-07

**Authors:** Anna Zabost, Sylwia Brzezińska, Monika Kozińska, Maria Błachnio, Jacek Jagodziński, Zofia Zwolska, Ewa Augustynowicz-Kopeć

**Affiliations:** ^1^Department of Microbiology, National Tuberculosis and Lung Diseases Research Institute, 01-138 Warsaw, Poland; ^2^Mazovian Center for Lung Diseases and Tuberculosis, 05-400 Otwock, Poland

## Abstract

Isoniazid (INH), a key agent in the treatment of tuberculosis (TB), is metabolized primarily by the genetically polymorphic N-acetyltransferase 2 (NAT2) enzyme. Patients treated with INH can be classified as fast, intermediate, and slow acetylators. The objective of this study was to explore the relationship between NAT2 genotypes and the serum concentrations of INH. Blood samples from 130 patients were taken for the analysis, and plasma INH concentrations were determined by using the high-performance liquid chromatography (HPLC) technology. Acetylation genotype was determined on genomic DNA by using an allele-specific polymerase chain reaction-restriction fragment length polymorphism (PCR–RFLP) assay. Once the NAT2 genotypes were established, patients were classified into three categories: fast, intermediate, and slow acetylators. Of the 130 patients studied, 84 (64.6%) were slow, 39 (30%) were intermediate, and 7 (5.4%) were fast acetylators. Analysis of INH concentrations in the blood of patients receiving the approximate doses of the drug revealed that, at the time intervals examined, the average concentration of INH was 2- to 7-fold higher among slow acetylators compared to fast and intermediate acetylators. *Conclusion*. Determining mutations in the NAT2 gene enabled the identification of the INH acetylation type in patients and the genotyping results were consistent with the phenotype determined by methods of measurement of drug bioavailability.

## 1. Introduction

Isoniazid (INH) is an essential component of all current chemotherapeutic regimens used for the treatment of tuberculosis (TB). INH is not only highly effective against *Mycobacterium tuberculosis*, but is also widely affordable, inexpensive, and well tolerated. The major pathway for metabolizing INH involves acetylation to acetylisoniazid by a noninducible hepatic and intestinal enzyme, N-acetyltransferase (NAT). The rate of acetylation is constant in every individual but varies between patients. The human population is divided into three different phenotypic groups according to acetylation rate, that is, slow, intermediate, and fast acetylators.

**Figure 1:**
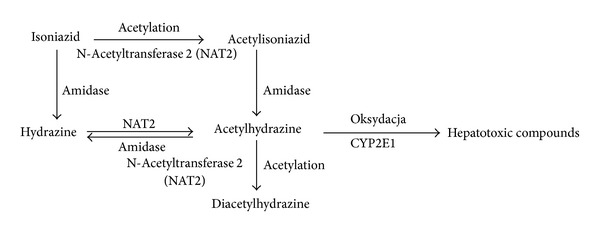
Schematic of the biotransformation of isoniazid [1].

INH is an easily digestible and well-absorbed drug, and maximum plasma concentrations occur 2 hours after oral intake. INH is metabolized internally to acetylisoniazid by NAT, which is then hydrolyzed to acetylhydrazine. Acetylhydrazine can be transformed into diacetylhydrazine by a process of acetylation or oxidized by cytochrome P-4502E1 (CYP2E1) to form hepatotoxic compounds. Low NAT activity increases the risk of hepatic damage because the majority of acetylhydrazine is oxidized [1, 2]. Depending on its serum concentration, INH can be both an inducer or an inhibitor of CYP2E1. High concentrations of INH repress the CYP2E1 activity, whereas low INH concentrations induce the enzyme [1]. The most severe hepatocellular damage during INH treatment is linked to an excess of hydrazine to which acetylhydrazine is hydrolyzed by amidase. The hepatotoxic potential of INH is greatly influenced by the enzymatic isoform of NAT. Low NAT activity (free isoform) increases the risk of hepatocellular damage due to oxidation of acetylhydrazine by CYP2E1 (Figure 1).

N-Acetyltransferase 2 (NAT2) exhibits genetic polymorphisms. Different acetylation phenotypes within a population are the result of mutations in the NAT2 gene. These mutations influence the activity (leading to either high or low activity) of the NAT enzyme (slow and fast acetylators, resp.) [3]. The NAT2 gene, located on chromosome 8p22, is autosomal dominant and intronless, with a single open reading frame of 870 bp. NAT2 enzyme detoxifies and inactivates drugs and xenobiotics in the liver. Polymorphisms of NAT2 confer slow, intermediate, or fast acetylator phenotypes with broad interethnic variations. There are currently known 53 NAT2 alleles, and each allelic variant reflects a combination of one, two, three, or four nucleotide substitutions. Within the coding region, there are seven missense mutations (G191A, T341C, A434C, G590A, A803G, A845C, and G857A) and four silent mutations (T111C, C282T, C481T, and C759A) [4]. The wild type NAT2*4 allele is associated with the fast acetylator phenotype and does not have any nucleotide substitutions. The acetylation phenotype can be predicted with 95% accuracy by genotyping [5].

The aim of this study was to explore the relationship between the NAT2 genotype and the INH acetylation phenotype.

## 2. Materials and Methods

The present study was conducted at the National Tuberculosis and Lung Diseases Research Institute on 130 adult TB patients treated at the Mazovian Center for Lung Diseases and Tuberculosis in Otwock, Poland, over a 2-year period (i.e., from 2006 to 2007). All patients received INH orally (200, 250, or 300 mg per day). Doses were calculated per kg of body weight. Patients were prohibited from ingesting food for 3 hours after INH intake.

Patient information, including name, age, and weight, was obtained, and blood samples were collected at 0, 1, 3, and 6 hours after administration of INH.

The study protocol was approved by the Internal Ethics Committee of the National Tuberculosis and Lung Diseases Research Institute. The aim of the study was fully explained to each patient, and the written informed consent was obtained from the participants.

### 2.1. Genotyping

Blood samples from 130 patients were drawn into sterile tubes (PAXgene Blood DNA, Qiagen), and total genomic DNA was extracted using the Blood DNA kit. To identify the three NAT2* mutations, C481T (NAT2*5), G590A (NAT2*6), and G857A (NAT2*7), polymerase chain reaction-restriction fragment length polymorphism (PCR-RFLP) was performed following the method described by Spurr et al. [6], with some modifications. Briefly, after the initial amplification, PCR products were digested with KpnI, DdeI, TagI, and BamHI. The NAT2*5 mutant allele was identified by the loss of a KpnI restriction site and the gain of a DdeI site, NAT2*6, by the loss of a TagI site, and NAT2*7, by the loss of a BamHI site. Restriction digests were separated by electrophoresis on 8% polyacrylamide gels.

Patients were classified into three groups: fast acetylators (homozygous for wild type NAT2*4 allele), intermediate acetylators (heterozygous for NAT2*4 and a mutant allele), and slow acetylators (a combination of mutant alleles).

### 2.2. Measurement of INH Concentration

*Chromatographic Method*. Plasma concentrations of INH were measured by high-performance liquid chromatography (HPLC), as described by Seifart et al. [7]. Plasma samples were deproteinized with trichloroacetic acid. After centrifugation at 10,000 RPM for 10 min, 200 *μ*L of the deproteinized sample was combined with 20 *μ*L water and 40 *μ*L 1% cinnamaldehyde. After 10 min at room temperature, the sample was eluted with solvent A (50 mM KH_2_PO_4_) and solvent B (acetonitrile-isopropanol, 4 : 1) on a *C*_8_ 250 mm column and detected at 340 nm. Two criteria were used to determine the INH acetylation phenotype: index of acetylation (*I*_3_) (boundary value of 0.65) [8] and Armstrong's criteria, which is the plasma concentration of INH 6 hours after administration of the drug (*C*_6_) (boundary value of 0.8 *μ*g/mL) [9].

### 2.3. Pharmacokinetic and Statistical Analysis

Pharmacokinetic parameters were determined using a one-compartment open model with the Pharm/PCS program using the INH concentrations determined in this study [10, 11]. The AUC (AUC_0–6_, the area below the concentration curve over time in *μ*g/mL/h) was calculated as the sum of triangles and trapezoids. The *K*_*e*_ (fixed speed of elimination 1/h) was calculated by a computer program, and the *T*_0,5_ (time of biological half-life in h) was determined by dividing 0.693 by *K*_*e*_. For statistical analyses, the paired Student's *t*-test was used, with statistical significance defined as *P* < 0.01.

## 3. Results

**Figure 2:**
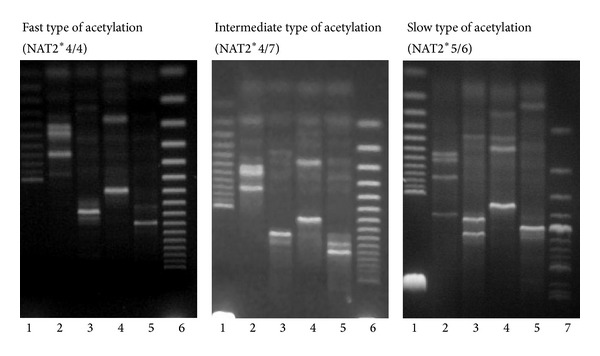
Identification of N-acetyltransferase 2 alleles. Restriction analysis of PCR product (lane 1: molecular size marker 25 bp, lane 2: Tag1, lane 3: Kpn1, lane 4: Dde1, lane 5: BamH1, lane 6: molecular size marker 50 bp, and lane 7: molecular size marker 100 bp).

There were 9 different NAT2 genotypes among the 130 TB patients examined. Seven (5.4%) patients with a NAT2*4/*4 genotype were classified as fast acetylators; 39 (30%) patients with a *4/*5, *4/*6, or *4/*7 genotype were classified as intermediate acetylators; and 84 (64.6%) patients with a NAT2*5/*5, *5/*6, *6/*6, *6/*7, or *7/*7 genotype were classified as slow acetylators (Table 1, Figure 2).

The mean plasma INH concentrations of homozygous wild type (wt/wt), heterozygous (wt/m), and mutant/mutant (m/m) groups were 1.2 ± 0.6, 2.2 ± 1.3, and 4.4 ± 1.5, respectively. The INH concentration in the m/m group was significantly higher than that in both the wt/m and wt/wt groups. Analysis of the correlation between genotype and plasma drug concentration revealed that the lowest concentrations of the drug were associated with the NAT2*4/4 genotype, whereas the NAT2*7/7 genotype, which is indicative of slow acetylation, was correlated with the highest INH concentrations (Table 1).

Examination of the acetylation type, as determined by genetic and chromatographic methods, demonstrated that the parameters that best differentiated between the three types of acetylation were AUC_0–6_, AUC_total_, and maximum concentration of INH (*C*_max⁡_).

For the pharmacokinetic parameters, *I*_3_, *C*_6_, *K*_*e*_, and *T*_0,5_, no statistically significant differences were observed between the fast (*I*_3_ = 0.36) and intermediate (*I*_3_ = 0.55) rates of acetylation as determined by genotyping. Significant differences for these indicators of bioavailability were, however, found between slow acetylation and the other two types of acetylation (fast and intermediate) (Table 2).

## 4. Discussion

The process of INH acetylation is well described, and its practical and theoretical aspects were systematically studied in the early years of INH use for the treatment of TB. In 1973, a report by the World Health Organization underscored the significance of determining a patient's acetylation phenotype during INH administration, as the drug is subject to biotransformation by NAT in the liver [12]. The way the INH is acetylated in the liver is polymorphic, and three types of individuals, namely, fast, intermediate, and slow acetylators, exhibit different plasma concentrations of the drug after the administration of the same dose. In addition, the rate of the elimination of INH is different between fast and slow acetylators [13].

The activity of the polymorphic enzyme NAT2 influences the metabolism of INH, the rate of its elimination from the body, and possibly the development of toxic side effects. Consequently, the rate of INH acetylation is a potential risk factor for liver failure. Acetylation polymorphisms are associated with interindividual variability in plasma concentration and half time of INH.

A fraction of INH is eliminated in its biologically active form, with the rest excreted as inactivated acetylated metabolites. Most of the INH (75–95%) is eliminated in the urine in a metabolized form within 24 hours of ingestion [14]. Depending on the acetylation type, 3–30% of INH is eliminated in an unaltered form [15]. These proportions also depend on the individual acetylation capacity. INH is partially eliminated in the feces, and some of the thus eliminated fraction is broken down into as yet unknown metabolites.

The results of this study demonstrate that differences in the dynamics of INH metabolism among slow, intermediate, and fast acetylators determine the concentration of the drug and its metabolites. Therefore, the bioavailability of the drug among fast and intermediate acetylators is much lower than it is among slow acetylators, and the anti-TB therapeutic range of INH is maintained for only a short period of time. Among slow acetylators, symptoms related to the accumulation and toxic effects of the drug occur more often. Consequently, slow acetylators should be monitored to avoid side effects [16–19].

Chromatographic methods of determining plasma concentrations of INH are precise and, compared to biological methods, involve a shorter waiting period (1 day). This method is useful for both scientific purposes and for monitoring TB patients undergoing treatment. This method allows to defining the type of acetylation and precisely monitor the changes in drug concentration. Published reports suggest that the therapeutic plasma concentration of INH should range from 1 to 2 *μ*g/mL during the first 3 hours after oral administration [20]. Here, we observed that four patients (9% of fast and intermediate acetylators) did not exhibit the proper INH concentration.

We described the variability in the pharmacokinetics of INH in TB patients after standard dosing, its demographic characteristics, and its relationship with genetic variations in NAT2. In addition, we clearly showed that the overall INH exposure is indirectly proportional to the number of highly active NAT2 alleles, consistent with findings from recent clinical trials. Carriers of the NAT2*4/*4 genotype (two high-activity NAT2 alleles) have been reported to exhibit lower INH concentrations at 3 h after dose than those with other NAT2 genotypes [21]. Parkin et al. suggested a link between the NAT2 genotypes, consisting of the recently defined 14 mutant alleles, and the acetylator phenotype in TB patients. These findings could pave the way to individualized dosage regimens. In addition, further sequence analysis of an expanded population may discover other mutations in the NAT2 gene. The main purpose of the present study was to clarify the applicability of genotyping as an alternative to therapeutic drug monitoring in the clinical setting.

Mutations in the NAT2 gene have been shown to account for the majority of the slow acetylator phenotypes among humans (NAT2*5, NAT2*6, and NAT2*7) [22]. Previous investigation of the functional characteristics of NAT2 single nucleotide polymorphisms has revealed that different molecular mechanisms underlie the slow acetylator phenotype and variations in the enzymatic activity of NAT2 between different alleles [23].

Patients with the NAT2*4/4 genotype (two high-activity NAT2 alleles) have been reported to exhibit lower INH concentrations than those with other NAT2 genotypes. Slow acetylators had two- to sevenfold higher INH concentrations at 3 and 6 h after drug administration than did fast acetylators [24].

## 5. Conclusions

The method of NAT2 genotyping is simple and fast. Therefore, it is suggested to be used as an alternative to therapeutic drug monitoring in clinical practice.

The data from the present study and other published reports clearly suggest that the determination of the NAT2 genotype prior to INH administration is clinically relevant for the prediction of pharmacokinetic variability and the possible adjustment of INH dosing regimens.

It must also be noted that INH will be predominantly employed in TB control programs in the developing world. Here, large numbers of patients must be managed by programs with limited resources. Under these circumstances, an individualized approach to INH dosing may not be feasible, and, thus, a compromise must be adopted.

## Figures and Tables

**Table 1:** The INH concentration at 3 hours after drug administration among various NAT2 genotypes.

Genotype	Number	%	INH (*µ*g/mL)	AUC_0–6_
NAT2∗4/∗4	7	5,4	1,2 ± 0,6	8,55 ± 4,6
wt/wt	7		1,2 ± 0,6	8,55 ± 4,6
NAT2∗4/∗5	25	19,2	2,3 ± 1,2	15,7 ± 6,2
NAT2∗4/∗6	13	10	2,1 ± 1,4	14,7 ±6,3
NAT2∗4/∗7	1	0,8	1,2	15
wt/m	39	30	2,2 ± 1,3	15,4 ± 6,1
NAT2∗5/∗5	22	16,9	4,1 ± 1,2	23,5 ± 6,1
NAT2∗5/∗6	45	34,6	4,2 ± 1,5	24,5 ± 7,6
NAT2∗6/∗6	10	7,7	4,4 ± 1,0	25,9 ± 5,8
NAT2∗6/∗7	5	3,8	4,6 ± 1,7	25,9 ± 7,8
NAT2∗7/∗7	2	1,5	7,7 ± 1,1	44,1 ± 7,1
m/m	84	64,6	4,4 ± 1,5	25,0 ± 7,5

AUC_0–6_: area under the concentration curve during the period of 0–6 hours

**Table 2:** Pharmacokinetic parameters of INH in plasma among fast, intermediate, and slow acetylators by the chromatographic method.

Genotype of acetylation	Pharmacokinetic parameters of INH						
	*I*_ 3_	*C*_ 6_	*K*_*e*_	*T*_ 0,5_	*C*_ max_	AUC_0–6_	AUC_total_
Fast	0,36^a^	0,27^a^	0,51^b^	1,59^a^	3,39^a^	8,6^a^	9,2^a^
Intermediate	0,55^a^	0,70^a^	0,41^b^	1,85^a^	5,80^b^	15,4^b^	17,5^b^
Slow	0,97^b^	2,20^b^	0,27^a^	3,14^b^	7,09^c^	24,5^c^	35,5^c^

The different letters in the columns indicate significant differences (at *P* ≤ 0.05) between the respective types of acetylation.

*I*_ 3_: index of acetylation.

*C*_ 6_: concentration of INH 6 hours after drug administration.

*K*_*e*_: fixed speed of elimination 1/h.

*T*_ 0,5_: elimination half time.

AUC_0–6_: area under concentration curve in the period 0–6 hours.

AUC_total_: area under concentration curve.
